# Supplementary material for: Inhibitory Mechanisms of Lusianthridin on Human Platelet Aggregation
Source: Int J Mol Sci. 2021 Jun 25;22(13):6846. doi: 10.3390/ijms22136846 (PMC8267677; doi:10.3390/ijms22136846)
Supplement: Supplementary file 1 [file ijms-22-06846-s001.zip › ijms-1249498-supplementary.pdf]

Supplemental Information For

Inhibitory Mechanisms of Lusianthridin on Human Platelet Aggregation

Hla Nu Swe, Boonchoo Sritularak, Ponlapat Rojnuckarin and Rataya Luechapudiporn

S1. Inhibitory effect of lusianthridin on the activity of cyclooxygenase enzymes

Lusianthridin exhibited a concentration-dependent inhibitory effect on both COX-1 and COX-2 enzymatic activities. The IC_{50} values of each inhibition were derived using variable slope non-linear regression using GraphPad Prism 9.1 (GraphPad, San Diego, CA, USA), and the graphs are depicted in Figure S1. According to the results, lusianthridin exerted a 50% inhibitory effect on COX-1 and COX-2 enzymes at the concentrations of 10.81 ± 1.12 µM and 0.17 ± 1.62 µM, respectively.

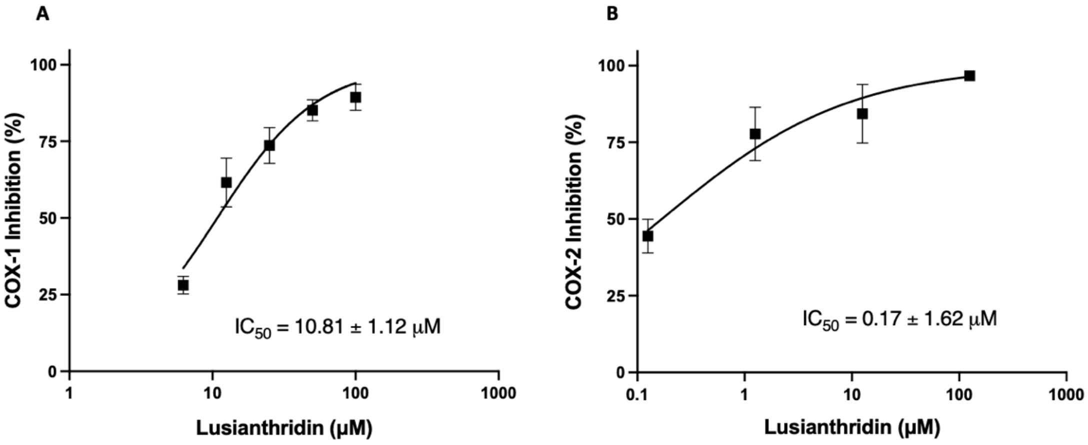

Figure S1: Lusianthridin concentration-dependently inhibited the expression of COX-1 (A) and COX-2 (B) enzymes. Data are expressed as mean ± SEM; n=3).
